# JAK/STAT Signaling and Cervical Cancer: From the Cell Surface to the Nucleus

**DOI:** 10.3390/genes14061141

**Published:** 2023-05-24

**Authors:** Arturo Valle-Mendiola, Adriana Gutiérrez-Hoya, Isabel Soto-Cruz

**Affiliations:** 1 Molecular Oncology Laboratory, Cell Differentiation and Cancer Research Unit, FES Zaragoza, National University of Mexico, Batalla 5 de Mayo s/n, Colonia Ejército de Oriente, Mexico City 09230, Mexico; arturo.valle@zaragoza.unam.mx (A.V.-M.); adrianagh85@hotmail.com (A.G.-H.); 2 Cátedra CONACYT, FES Zaragoza, National University of Mexico, Mexico City 09230, Mexico

**Keywords:** JAK/STAT pathway, signaling, cervical cancer, HPV

## Abstract

The Janus kinase (JAK)/signal transducer and activator of transcription (STAT) signaling pathway constitutes a rapid signaling module from the cell surface to the nucleus, and activates different cellular responses, such as proliferation, survival, migration, invasion, and inflammation. When the JAK/STAT pathway is altered, it contributes to cancer progression and metastasis. STAT proteins play a central role in developing cervical cancer, and inhibiting the JAK/STAT signaling may be necessary to induce tumor cell death. Several cancers show continuous activation of different STATs, including cervical cancer. The constitutive activation of STAT proteins is associated with a poor prognosis and overall survival. The human papillomavirus (HPV) oncoproteins E6 and E7 play an essential role in cervical cancer progression, and they activate the JAK/STAT pathway and other signals that induce proliferation, survival, and migration of cancer cells. Moreover, there is a crosstalk between the JAK/STAT signaling cascade with other signaling pathways, where a plethora of different proteins activate to induce gene transcription and cell responses that contribute to tumor growth. Therefore, inhibition of the JAK/STAT pathway shows promise as a new target in cancer treatment. In this review, we discuss the role of the JAK/STAT pathway components and the role of the HPV oncoproteins associated with cellular malignancy through the JAK/STAT proteins and other signaling pathways to induce tumor growth.

## 1. Introduction

Cervical cancer is the fourth cancer with the highest incidence and mortality worldwide, and its prognosis continues to be poor, especially when the disease is detected at a late stage. In 2020, GLOBOCAN reported 604,127 cervical cancer cases and 341,831 deaths, with a high prevalence in the last five years [1,2]. Treatment options for patients with cervical cancer vary depending on the stage at which they are detected but still are limited. For example, stage IA1 can be treated with conization or hysterectomy. Stages IA2, IB, and IIA can receive treatments that include radical hysterectomy and lymphadenectomy, and patients with local or advanced cancer are treated with cisplatin-based chemotherapy. However, the risk of relapse is 10–20% for early phases of the disease and 50–70% for advanced phases of the disease. Nevertheless, cisplatin has a response rate of 13% (monotherapy) and 36% with dual therapy; for this reason, new strategies are generated combining chemotherapeutic drugs with monoclonal antibodies that recognize molecules that are essential for tumor growth, such as antibodies anti-vascular endothelial growth factor (Bevacizumab), which improves the response rate by up to 48%. However, the options are limited in the case of relapses, which makes it necessary to look for new therapeutic targets to improve the prognosis in patients with advanced disease [3]. Vaccination has helped to reduce cervical cancer prevalence. Three prophylactic vaccines for the human papillomavirus (HPV) have been approved, although they do not have therapeutic effects on existing infections: Gardasil^®^ (quadrivalent vaccine against HPV16, HPV11, HPV16, and HPV18), Cervarix^®^ (bivalent vaccine against HPV16 and HPV18), and Gardasil 9^®^ (nonavalent vaccine against HPV6, HPV11, HPV16, HPV18, HPV31, HPV33, HPV45, HPV52, and HPV58). It is clear that cervical smears and HPV vaccination have helped reduce cases in developed countries; however, cervical cancer still represents a public health burden affecting middle-aged women, mainly in countries with low incomes [2,4]. Persistent high-risk papillomavirus (hrHPV) infection is the leading cause of cervical cancer development; the most common hrHPV types are 16, 18, 31, 33, 35, 45, 52, and 58. Among them, two types (HPV16 and HPV18) cause between 70 and 72% of invasive cervical cancers [5]. hrHPV has two oncoproteins, E6 and E7, necessary for establishing and development of cervical cancer. Regulatory proteins pRB and p53 are the best-known targets of E6 and E7. However, there are other targets: E6 and E7 also regulate epigenetic marks, splice changes, and generate regulatory RNAs—regulators of the transcription—among other changes that allow the virus to proliferate in an uncontrolled manner and generate cell transformation and carcinogenesis [6,7,8,9]. After HPV infection, tumor development does not progress uniformly; precancerous lesions may relapse without any treatment for uncertain reasons [10]. Regardless, the constant HPV infection triggers molecular changes that silence the cell-mediated immune responses blocking clearance of the infection and elimination of abnormal cells [11]. However, a hrHPV infection is considered a necessary but insufficient cause for cervical cancer development [12]. There are different risk factors, such as initiation of sexual activity at an early age, sexual multiparity, vaginal irrigation or sexual intercourse, and biological factors, such as bacterial vaginosis [13,14], malnutrition, or sexually transmitted infections [15] that alter the vaginal microenvironment and contribute to the persistence of an HPV infection [16]. 

The JAK/STAT pathway induces the expression of critical mediators of cancer and inflammation, and dysregulation of this pathway is associated with several cancers. Persistent activation of different STATs is shown in various cancers, including cervical cancer, and the overactivation may be related to a poor prognosis and poor overall survival. The oncoproteins E6 and E7 play a central role in cervical cancer progression and may mediate the activation of the JAK/STAT signaling pathway [17,18]. In this review, we describe the JAK/STAT main players, its relationship to cervical cancer activation, and the crosstalk with other signaling pathways associated with HPV oncoproteins and cancer development.

## 2. The Discovery of the JAK/STAT Pathway

In recent years, an emergent field of investigation has been signal transduction pathways; this field has multiple applications, particularly in developing specific inhibitors for controlling the aberrant activation of some signal transduction pathways. 

The destiny of cells is mainly determined by the intracellular signaling pathways that regulate mechanisms involved in responses to ligands. These pathways are frequently activated through cell membrane receptors that bind to their cognate ligands to promote the mechanisms involved in controlling a wide variety of phenomena such as proliferation, apoptosis, hematopoiesis, tissue repair, adipogenesis, inflammation, and metabolic changes, among others [19]. 

The JAK/STAT pathway is well-conserved in metazoan cells [20]; several reports include more than 50 cytokines and growth factors that participate in the JAK/STAT signaling; for example, interleukins (ILs), interferons (IFN), colony-stimulating factors (CSF), and hormones [21]. At the beginning of the twentieth century, some studies associated mutations in JAK and STAT proteins (resulting in persistent pathway activation) with several disorders [22] and, moreover, the loss of JAK/STAT components with different diseases in humans; for example, severe combined immunodeficiency (SCID) [23].

The discovery of the JAK/STAT pathway dates back to 1989; however, some authors track its origin to 1950 [22]. JAK1 and JAK2 were described in 1989, and the first protein of the JAK family was cloned in 1990 [24]. The gene was named Tyk2; the most attractive characteristic of this molecule was the presence of a kinase-like domain next to the protein tyrosine kinase domain [25]. Because of this feature of the protein having one kinase-like domain next to the tyrosine kinase domain, or “two faces”, the name (JAK or Janus kinases) was derived from the Roman God of doorways with two faces, Janus. The protein functions with one domain including the kinase activity and a second domain that negatively regulates the kinase activity [22,26]. In 1992, Harpur et al. cloned the DNA sequence that was complementary for JAK2 and showed that this protein had a similar structure, including both a kinase domain and the kinase-like domain (“pseudo-kinase” domain) [27]; in the same year, Shuai et al. reported cDNA clones named the signal transducer and the activator of transcription (STAT1 isoforms α and β, at that time known as STAT91 and STAT84) and STAT2 (or STAT113) [28,29]. Posteriorly, Darnell et al. obtained the clones for STAT3 and STAT4 from a lymphocyte cDNA library, demonstrating that IL-6 and EGF induced the phosphorylation of STAT3 [30,31]. The gene named mammary gland factor (MGF), encoding STAT5, was cloned and sequenced by Wakao et al. in 1994 [32]. The relationship between the proteins is revealed in the in the JAK/STAT pathway data from the early 90s. In 1992, Velazquez et al. showed that TYK2 is required in the IFN-α/β signaling pathway [33]; in 1993, Müller et al. demonstrated that IFN-dependent signaling needed JAK kinases to activate STAT proteins [34]. In later years, different elements and functions of the JAK/STAT signaling pathway were elucidated. Intensive research on the JAK/STAT signaling pathway function has continued, making the JAK/STAT an imperative target for research, along with the search for specific inhibitors to treat different diseases.

## 3. The JAK/STAT Pathway

**Figure 1:**
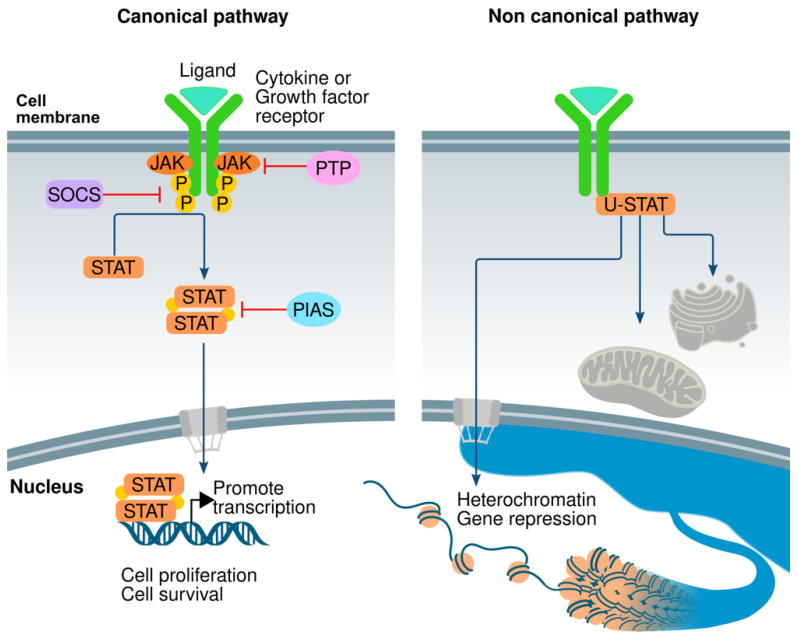
The JAK/STAT pathway players. In the canonical pathway, the ligand binds to its receptor to activate JAKs that phosphorylate specific TYR sites in the receptor to interact with STAT proteins. Then, JAKs phosphorylate STATs, forming protein dimers through SH2 domains to translocate into the nucleus to promote gene transcription to induce cellular responses such as proliferation or survival. In the non-canonical pathway, the non-phosphorylated STAT proteins (U-STAT) can go to the mitochondria or Golgi apparatus to control metabolic reactions. Moreover, they can enter the nucleus to repress gene transcription.

The canonical pathway initiates when the ligand binds to its cognate receptor to induce dimer or trimerization; however, some receptors, such as gp130 [35], erythropoietin receptor (EpoR) [36,37], TNF-R1 [38], IL-17R [39], IL-10R [40], and Growth Hormone receptor (GHR) [41], among others, can form inactive dimers before binding to the ligand, thus facilitating a rapid activation of different molecules that participate in the cellular response. The union between the receptor and its ligand induces transphosphorylation of JAKs, which activates them; these activated JAKs phosphorylate specific tyrosine residues of the receptor, originating docking sites for STATs. At these sites, JAK phosphorylates specific tyrosine residues of the STAT protein, which separates from the receptor and forms homo or heterodimers by an interaction between the SH2 domain of one STAT and a specific phosphotyrosine residue of the other STAT. These dimers translocate to the nucleus, where they can regulate the transcription of specific genes (Figure 1) [20,22,42,43]. Nonetheless, the JAK/STAT pathway is also involved in the non-canonical signal transduction pathway. In the canonical JAK-STAT interaction, there is a specific correspondence between the activity of the STAT protein and its tyrosine phosphorylation; nevertheless, unphosphorylated STAT (U-STAT) has relevant functions such as control of the metabolism in different cell compartments and specific control in mitochondria or Golgi apparatus [44,45,46,47,48,49,50,51]. U-STAT1 was necessary for the TNF-mediated apoptosis of U3A cells; however, the phosphorylation of the S727 at the C-terminal is required for this activity [52]. Some of the U-STAT pool is associated with the heterochromatin in the nucleus, in particular with heterochromatin protein-1 (HP1). It has been shown that STAT92E associates with HP1 to keep the structure of heterochromatin and gene repression (Figure 1) [53,54]. JAK or other kinases activate STAT proteins and induce HP1 to separate from the heterochromatin; then, phosphorylated STAT binds to specific sites on chromatin to change the structure to euchromatin and regulate gene transcription. This non-typical JAK/STAT signaling is necessary for maintaining the stability of heterochromatin [54,55,56]. In mammals, U-STAT5A binds to HP1α to keep the stability of heterochromatin to STAT92E in order to repress the genes involved in cancer development [57]. Another function of U-STAT5 is repressing the transcriptional program necessary for megakaryocytic differentiation by preventing the interaction of the transcription factor ERG (ETS-related gene), thus acting as an antagonist of the biological activity of pSTAT5 [58]. U-STAT3 competes with IκB for the union with non-phosphorylated NFκB, translocating to the nucleus and activating several NFκB-dependent genes [59]. U-STAT6, in addition to p300, binds to a consensus STAT6 binding site in the promoter of the Cox-2 gene to regulate its constitutive expression [60]. 

The understanding of the mechanism used by the U-STATs to translocate into the nucleus awaits further analysis. The nuclear export rate keeps most U-STATs in the cytoplasm at a steady-state; some of these molecules may be confined in the nucleus by DNA or chromatin association. There are other possible mechanisms which do not include U-STATs, such as the interaction with different transcription factors such as Interferon Regulatory Factor 1 (IRF1) or direct association with the nuclear pore complex [61,62,63].

JAK proteins can also be activated by tyrosine kinases with tumorigenic activity that do not interact with cytokine receptors [64]. It has been reported that the v-Abl (Abelson murine leukemia virus) can affect the JAK/STAT pathway by interfering with the association between suppressors of cytokine signaling (SOCS)-1 and JAK proteins [65]. The BCR-ABL, another tumorigenic kinase produced by the translocation of chromosomes 9 and 22, can exert anti-apoptotic effects and synergize with different hematopoietic growth factors maintaining the active form of JAK proteins through permanent phosphorylation, which helps to regulate STAT activation [66]. STATs can be phosphorylated by different non-receptor tyrosine kinases; for example, the tyrosine kinase c-Src phosphorylates STAT3, which increases tumor-related gene expression [67]. Moreover, STATs can be directly activated by other receptors that do not signal through JAK proteins. The epidermal growth factor receptor (EGFR) can activate STAT1, STAT3, and STAT5; in the same way, the platelet-derived growth factor receptor (PDGFR) directly activates STAT5 [68,69,70]. 

The JAK/STAT pathway crosstalks with different signaling pathways. In 2007, Levine et al. showed the role of JAK2 in myeloproliferative neoplasms in which the PI3K/Akt and the Ras/Raf/MAPK/ERK signaling pathways were activated [71]. In the transforming growth factor-β (TGFβ) pathway, STAT3 forms active molecular complexes to induce astrocyte differentiation. For example, STAT3 interacts with SMAD1 forming a complex through p300, leading to cell differentiation. After TGFβ stimulation, JAK 1 activates STAT3 in a SMAD-independent manner. Moreover, STAT3 is activated after TGFβ binding in a SMAD-dependent manner, including the participation of JAK1. Activated STAT3 and SMAD bind to their respective DNA sequences in the JUNB promoter to increase the expression of genes related to TGFβ responses [72]. Moreover, STAT3 attenuates the interaction between SMAD3–SMAD4 to form a complex and suppresses SMAD3-DNA binding [73]. The phosphorylation state of STAT3 and SMAD3 determines the function of the protein complex that can cooperate or antagonize the response [72].

IL-6 is an essential ligand which connects the NF-κB signaling pathway with STAT3, which has a central function in the activation of the NF-κB pathway. STAT3, when constitutively active, drives hyperacetylation of RelA, depending upon the presence of p300. Thus, NF-κB remains in the nucleus and promotes the activation of NF-κB in transformed cells and tumor-related hematopoietic cells [74].

## 4. Players of the JAK/STAT Signaling Pathway

### 4.1. Cell Surface Receptors

JAK/STAT signaling initiates when JAK proteins are activated after ligand binding (for example, interleukins, growth factors, or interferons) to specific cell membrane receptors. Many transmembrane receptors have been related to the JAK/STAT pathway activation; the most common receptors associated with the JAK/STAT signaling pathway are the cytokine receptors [19,20,22]. 

Cytokine receptors initiate the JAK/STAT signaling pathway through a diverse combination of different JAKs and STATs, giving such a versatile nature to this pathway. The most common receptors that can trigger the JAK/STAT pathway are interleukin receptors (ILR), interferon receptors (IFNR), and colony-stimulating factor receptors (CSFR). The receptors for interleukins 2, 3, 4, 6, 7, 9, 10, 11, 12, 13, 15, 20, 21, 22, 23, 27, 31, and Leptin and gp 130 subunit activate specific members of the JAK family. JAK1 functions as a common factor among the JAK proteins but there are many combinations of downstream effectors [75]. For instance, heterodimerization of the IL-2Rβ subunit and γ common subunit (γc) through their cytoplasmic domains activates JAK1 (associated with IL-2Rβ) and JAK3 (associated with γc) [76]. When IL-2 binds to its receptor, it is followed by the activation of STAT5 in cervical cancer cell lines [77,78]. An essential function of IL-2 and its receptor has been reported in breast and cervical cancer growth, correlating malignancy of the tumor and expression of this receptor [77,79,80,81]. 

The erythropoietin receptor (EPOR) shares extra-cellular structure arrangements with the cytokine receptor family; for example, EPOR and IL-2Rβ share 45% amino acid similarity in their cytoplasmic regions [82] and can induce a rapid JAK2 phosphorylation which depends on the dose [83]. EPOR is expressed in cervical cancer, and the binding of erythropoietin (Epo) to its receptor induced cell proliferation; such cell response was related to the activation of JAK2, JAK3, STAT3, and STAT5, but not JAK1 and STAT1 in cervical cancer [84].

The cytokine receptors IL-4R [85] and IL-13R [86] send signals through STAT6 and activate the transcription of a different set of genes to synthesize proteins necessary for T cells proper function in contrast to non-lymphoid cells [87]. 

G protein-coupled receptors (GPCR) can also activate JAKs [88]. Among GPCRs, the chemokine receptor CXCR4 has a central role in cancer cell growth. The CXCR4 activates in response to stromal cell-derived factor (SDF-1alpha). JAK2 and JAK3 phosphorylate specific tyrosines of CXCR4 to recruit multiple STATs and activate them by tyrosine phosphorylation [89]. Other GPCRs with the ability to activate the JAK/STAT pathway are platelet-activating factor receptor (PAFR), angiotensin II receptor type 1 (AT1R), and bradykinin B2 receptor (B2R), all of which activate TYK2 (including JAK2 for PAFR and AT1R) to initiate the JAK/STAT pathway [75].

Some tyrosine kinase receptors (TKR), such as EGFR, induce STAT1 phosphorylation, initiating the complex formation of STAT1 and STAT3 with JAK1 and JAK2, respectively [90]. Other receptors, such as vascular endothelial growth factor receptor (VEGFR), fibroblast growth factor receptor (FGFR), and PDGFR, have also been related to the JAK/STAT pathway. FGFR stimulates STAT1/STAT3 through JAK2 [91]; the activation of STAT3 by tyrosine phosphorylation induced by this receptor is JAK-dependent, forming a complex between JAK2, c-Src, and the receptor FGFR1 [92]. Zhao et al. have shown that VEGFR-2 recruited JAK2 and STAT3 to initiate the JAK2/STAT3 signaling pathway, leading to over-expression of MYC and SOX2 [93].

Some reports indicate that Toll-like Receptors (TLRs) could stimulate STAT3 activity; this pathway plays a role in tumor development induced by these receptors [94]. Nevertheless, their involvement is controversial. For example, overexpression of TLR4 leads to increased STAT3 activity in epithelial cells of the gut, which is associated with the clinical results of colon adenocarcinoma [95]. Moreover, TLR4 upregulates IL-6 in lymphomas [75,96]. Furthermore, it was proposed that JAK2 is activated by TLR9 through Frizzled 4, resulting in the phosphorylation of STAT3 [97].

### 4.2. JAKs

**Figure 2:**
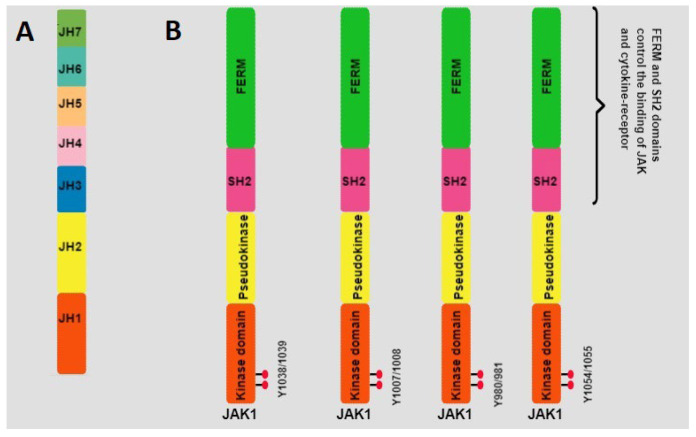
JAK proteins structure. The seven JH domains are indicated in A. The currently accepted domains are shown in B. Red circles represent the tyrosine residues involved in the regulation of enzymatic activity.

The JAK family includes four members: JAK1, JAK2, JAK3, and TYK2. These JAK proteins have a similar structure which consists of seven domains that share a high homology (the JAK homology domain, JH). JH1 is the first JH at the carboxyl terminus that contains the kinase domain, formed by approximately 250 amino acids. JH2 is the pseudokinase (PK) domain; JH2 and the kinase domain have a similar structure, but JH2 does not have kinase activity. The main function of the PK domain is to control the tyrosine kinase activity by binding to the kinase domain. The PK domain also participates in the JAK-STAT interaction. JH3 combines with the JH4 domain and integrates the Src-homology 2 (SH2) domain. The FERM (four-point-one, ezrin, radixin, moesin) domain is formed by combining the JH5, JH6, and JH7 domains. An important function of SH2 and FERM domains at the amino-terminal end is to control the specific interaction between JAK and cytokine-receptor membrane-proximal box1/2 motifs (Figure 2) [98,99,100,101]. 

The conserved tyrosines Y1038/Y1039 in JAK1 constitute an essential motif in the activation loop. Phosphorylation of both tyrosine residues in the kinase domain of each JAK protein generates a very stable conformation for substrate interaction [102]. JAK1 is broadly expressed in different cell types and can phosphorylate all STAT proteins [42]. Three cytokine-receptor families activate JAK1: Cytokine receptors with the γ common (γc) receptor subunit (also known as IL-2 family receptors) such as IL-2R, IL-4R, IL-7R, IL-9R, and IL-15R receptors; class II cytokine receptors, which include the IFNα/βR, IFN-γR, and IL-10 family cytokine receptors; and cell membrane receptors that contain the gp130 subunit, such as the IL-6R, IL-11R, oncostatin M (OSM) receptor, cardiotrophin-1 CT-1) receptor, ciliary neurotrophic factor (CNTF) receptor, and leukemia inhibitory factor (LIF) receptor [103,104]. JAK2 has two conserved tyrosine residues, Y1007 and Y1008 [102]. JAK2 can be activated by different components of the class II cytokine receptor and gp130 receptor families. Furthermore, JAK2 participates in the transduction of signals initiated by some members of the IL-3 receptor family (such as IL-3R, IL-5R, and GM-CSF receptor) and other receptors such as growth hormone receptor (GH), prolactin receptor, erythropoietin receptor (EPO), and thrombopoietin (TPO) receptor, which have only one transmembrane chain [105]. The two conserved phosphorylation sites in JAK3 are Y980 and Y981 [102]. JAK3 activates the signal transduction of the receptors that share the γ common subunit (γc); for example, IL-2R, IL-4R, IL-7R, IL-9R, IL-15R, and IL-21R [106]. In the case of Tyk2, Y1054/Y1055 are conserved phosphorylation sites [101] and can transmit IFN-α/β signals [107]; also, it initiates signals by IL-6, IL-10, IL-12, IL-13, and IL-23 [108,109,110,111].

### 4.3. STATs

The STAT family comprises seven different proteins—STAT1, STAT2, STAT3, STAT4, STAT5a, STAT5b, and STAT6—that have a common structure with highly conserved domains. STATs consist of 750–900 amino acids forming defined domains that give structure and function to the protein: the amino-terminal domain, coiled-coil domain, DNA-binding domain, linker domain, SH2 domain, and Carboxy-terminal domain, including the transcriptional activity; these domains control all the different activities of STATs [30,31,112,113,114,115,116]. A complete review of the detailed structures of STATs has been published [117]. Here, we briefly describe the different STAT domains. The N-terminus, formed by approximately 50 amino acids, participates in the formation of STAT dimers, which translocate to the nucleus. Some reports have shown that the N-terminal domain promotes the interaction of STATs with transcription co-activators of the protein-inhibitor of activated STAT (PIAS family) and regulates nuclear translocation [118,119,120,121]. The structure of the coiled-coil domain comprises the four-helix bundle and is associated with regulatory proteins; its function is to control nuclear import and export processes. The sequence of the coiled-coil domain is the most variable between different STATs. This domain can interact with different regulators such as Nmi, c-Jun, and p48/IRF9, among others [122,123,124,125,126,127,128]. Next to the coiled-coil domain is the DNA-binding domain, which binds to DNA sequences in the regulatory region of the target gene. Moreover, it regulates nuclear import and export [129,130]. This domain regulates STAT selectivity for DNA recognition and participates in anti-parallel dimer contacts [21]. All STAT proteins can bind to DNA except STAT2, which only binds to DNA when it interacts with STAT1 to form a heterodimer. There is a consensus sequence for STATs, TTNNNNNAA, where N represents any nucleotide. There is an optimal DNA binding sequence reported for each STAT. The sequence for optimal DNA binding for STAT2 differs from the other members of the family; this difference could explain why STAT2 monomers cannot bind DNA. This sequence is related to a specific amino acid sequence in each STAT protein that can bind to DNA (Table 1) [131]. The differences in binding affinities for each STAT protein confer specificity to gene activation mediated by STATs. [21]. 

The linking domain functions as a connector between the DNA-binding domain and the SH2 domain. The linking domain regulates the transcriptional activity of STAT1 and nuclear export and dimerization [124,132]. The SH2 domain is highly conserved in the STAT family [133]; however, the tertiary structure of this domain is the most variable. The main function of the SH2 domain is to recognize phosphotyrosine motifs in cytokine receptors. Moreover, the SH2 domain helps an active JAK to induce the interaction of the SH2 domain of one STAT monomer with the tail of another STAT monomer after tyrosine phosphorylation to form a functional homodimer or heterodimer [134,135,136,137]. The C-terminal domain, which includes the transcriptional activation domain, is necessary to bind to DNA transcription elements and recruit co-activators by a conserved serine phosphorylation site to regulate transcription and protein stability. This domain is highly variable and contains a serine residue that is phosphorylated to enhance transcriptional activation. [20,22,75]. STAT proteins have different activities, but their molecular function can be redundant. These different activities and pathway interactions can be explained by their functional structure. All STAT members have a similar polypeptide structure, which can be described as one single polypeptide with a tri-dimensional structure consisting of six domains with specific secondary and tertiary structures [138]. This characteristic structure of the STAT family derives from the gene structure. The genomic structure of STATs has a complex organization with a large number of exons. For example, there are 24 exons in Stat1, Stat2, and Stat3 genes [139,140]. Transcription of Stat genes can produce spliced transcripts coding for multiple STAT variants. Some of these variants are truncated at the carboxy-terminal; for example, STAT1b1, STAT3b, STAT5a-2, and STAT5b-2. These truncated proteins lack some essential tyrosine or serine residues; therefore, their activation varies, and they can have different transcriptional activities on target genes [141,142]. STAT proteins share the six domains, but the differences within these domains define the specific activity of each member of the family, and the differences are also present in the genes; thus, the gene structure defines the protein domains.

STAT1 has two splice variants: STAT1α, a 91 kDa protein, which shares similar characteristics to other members of the STAT family; this splice variant has a complete C-terminal domain because it has two phosphorylation sites (position 701 and 727); and STAT1β, which has a size of 84 kDa and only one phosphorylation site (701) [143]. STAT1β lacks most of the transcription-activation domain and the serine 727 phosphorylation site at the C-terminus; consequently, its functionality is reduced [144]. It has been shown that IFN-type 1 ligands activate STAT1β, but its response to IFN-γ is deficient [104]. STAT1 is activated by IFN, IL-2, IL-6, PDGFR, EGFR, hepatocyte growth factor (HGF), tumor necrosis factor (TNF), and angiotensin II. The function of STAT1 is diverse; alongside cell growth inhibition [145,146], it regulates cell differentiation [147,148], promotes cell apoptosis [149,150,151], inhibits tumor occurrence [152], and is involved in the antigen presentation processes depending on the major histocompatibility complex (MHC) [153]. In cervical tissues, overexpression of STAT1 mRNA and protein have been reported in cervical cancer samples compared to normal cervical tissues [154]. In 2011, Rajkumar et al. observed that STAT1 was overexpressed in cervical intraepithelial neoplasia (CIN) 1 and 2 as well as in invasive cancers; however, in CIN3 and cervical carcinoma in situ (CIS), there was a decrease in STAT1 expression [155]. In 2020, Yi et al. reported the overexpression of STAT1 in CIN1, CIN2, CIN3, and cervical cancer, but this overexpression did not significantly affect overall survival [156]. However, STAT1 was not determined in its phosphorylated form in these studies. STAT1 could play an important role by increasing the sensitivity of cervical tumor cells to chemotherapy drugs and radiation. Buttarelli et al. reported that high levels of STAT1 are expressed in patients with cervical cancer sensitive to chemoradiation compared to those resistant to chemoradiation treatment [157]. Since 99.7% of cervical cancer cases are the result of persistent genital HPV infection, it is important to mention the effect of the virus and its oncoproteins on the JAK/STAT pathway. For example, it has been observed that the expression of the hrHPV E6/E7 oncoproteins, individually or together in keratinocytes, induces a decrease in the expression of STAT1α/β [158,159,160]. Viral oncoproteins can affect the signaling cascade for STAT1 activation at different levels; it has been reported that HPV18 E6 can bind to Tyk2, avoiding its interaction with IFNAR1 and thus preventing STAT1 phosphorylation and inhibiting the IFN-α pathway [161]. It has also been observed that HPV16 E6 and E7 alone or together diminish the expression of STAT1, its translocation to the nucleus, and its union to ISRE elements [162]. The involvement of STAT1 could be necessary for the viral cycle; it is reported by Hong et al. that the decrease of STAT1 is required for the amplification of the viral genome at the beginning of the infection, probably to suppress the genes inducible by interferon and evade the immune system and achieve an effective infection [160].

STAT2 cannot bind to DNA and cannot form homodimers despite possessing all six complete domains [163]. STAT2 differs from other STATs since it is the only member of the STAT family that does not interact with the original γ-activated site in the DNA. STAT2 activates in response to type I interferons, including IFN-α and IFN-β. The known biological functions include antiviral effects and immune regulation [164,165]. The role of STAT2 in cervical cancer is poorly studied, a higher expression of STAT2 has been observed in adenocarcinoma samples compared to normal tissue. In samples with CIN, there is a higher expression of STAT2 compared to samples from patients with cervicitis; however, no correlation was found between the expression of STAT2 and the severity of the lesion. In the 5-year survival analysis, no significant differences were observed between patients with positive samples for STAT2 versus negative samples for STAT2 [166]. The effect of HPV and its viral oncoproteins on STAT2 is also poorly understood and what we know is that the response given by STAT2 is due to the formation of a heterodimer with STAT1. For example, as previously mentioned, the HPV-18 E6 oncoprotein can physically interact with Tyk2 to inhibit its interaction with IFNAR1, thus affecting the formation of phosphorylated STAT1 and STAT2 heterodimers, their binding to IRF9 (preventing the formation of the ISGF3 complex), and its translocation to the nucleus [161,167]. These mechanisms affect the response to IFN type I. 

STAT3 has two splicing variants, STAT3α and STAT3β, with remarkable structural differences. At the C-terminal, STAT3α has a full-size domain, while STAT3β is missing 55 amino acids, replaced only by seven amino acid residues, and both have different functions [168,169]. STAT3 activates when the specific phosphosites are phosphorylated: either Y705 or S727; however, STAT3β only activates when Y705 is phosphorylated. This splice variant lacks the other activating residue S727. Due to the absence of S727, STAT3β has a better specific DNA-binding activity than STAT3α, but STAT3α is better at inducing transcription [169,170]. STAT3 activates in response to ligands of the IL-6 family: IL-10 family, IL-21, IL-27, G-CSF, leptin, IFN, and IL-2 [30,68,171,172]. STAT3 is mainly involved in multiple effects cell growth, differentiation, and apoptosis [173]; regulation of the immune response and tumor occurrence and metastasis [174,175,176,177]; regulation of tumorigenesis [172,178,179,180]; and regulation of cancer stem cells (CSCs) [181]. The expression of STAT3 in cervical cancer has acquired great relevance due to various reports that associate its activity with the malignancy grade of cervical lesions [182,183,184,185,186]. There is a close relationship between STAT3 and high-risk HPVs. In cervical cancer cell lines, it has been observed that HPV-positive lines have higher levels of STAT3 and pSTAT3 compared to HPV-negative cell lines [183,187,188]. In addition, an association has been found between pSTAT3 levels with high HPV genome copy numbers and viral genome integration [189]. STAT3 is a transcription factor that has a central role in tumor development. Various studies show that its inhibition decreases cell proliferation, favoring its sensitivity to chemotherapeutic drugs, increasing autophagy levels, and inhibiting the capacity of tumor cells to metastasize and tumorigenesis [182,190,191,192,193]. The E6 and E7 viral oncoproteins are also widely related to STAT3 and its phosphorylation. It has been shown that STAT3 inhibition in tumor cells decreases the expression of E6 and E7 proteins. On the contrary, transfection of C33A (HPV-) cells with E6 or E7 positively regulates STAT3 expression and its phosphorylation. However, the HPV18 E6 protein affects serine and tyrosine phosphorylation of STAT3, which generates the fully active form of this signal transducer [190]. Another strategy tumor cells use to keep STAT3 active is the production of large amounts of IL-6, which is used in an autocrine manner to signal via its receptor (IL6R) and maintain sustained STAT3 phosphorylation [188]. STAT3 is a central player in cancer because it controls the transcription of genes involved in the cell cycle, survival, metabolism (Warburg effect), epithelial–mesenchymal transition, chemoresistance, immunosuppression, angiogenesis, migration, and invasion. For this reason, a large number of studies analyze the molecular mechanisms to inhibit STAT3 to use them as therapeutic targets in cervical cancer [17].

STAT4 activates in response to type I interferons: IL-12 and IL-23 [194,195]. STAT4 is fundamental in differentiating and developing Th1 cells and helper T cells. Moreover, STAT4 participates in the response of the germinal centre [196]. The role of STAT4 in cervical cancer has been poorly studied and understood; however, a higher expression of STAT4 has been reported in histological sections of patients with squamous cell carcinoma and adenocarcinomas compared to non-cancerous lesions. In addition, the expression of STAT4 correlated with metastasis to lymph nodes, which could indicate an association between the expression of STAT4, the process of tumorigenesis and metastasis [197]. Furthermore, the study with K14E7 transgenic mice showed increased STAT4 gene expression in the skin of the mice, suggesting a likely regulation by the E7 oncoprotein [198].

STAT5 includes STAT5a and STAT5b, and both show a 91% amino acid homology. STAT5a comprises 794 amino acids, while STAT5b comprises 787 amino acids [85,199]. There are reports showing that STAT5a form dimers, but also can form tetramers. On the contrary, STAT5b only form dimers to bind to DNA [200]. STAT5 is activated by cytokines such as IL-3, prolactin, the IL-2 cytokine family, EGF, EPO, GM-CSF, TPO, GH, and PDGF [31,85,116,199,201]. STAT5 is capable of performing the following functions: regulation of growth and development [202,203], regulation of the immune system [200,204], regulation of tumor immunity [202,205], and regulation of cell growth, differentiation, and apoptosis [206,207]. A high expression of STAT5 has been reported in cervical tumour lesions and cancerous tissues compared to samples from the cervix without lesions. In addition, there is a correlation between STAT5 phosphorylation and the degree of cervical lesion. HPV-positive cervical cancer cell lines express higher levels of STAT5 [208,209]. Thus, high-risk viruses and their viral oncoproteins also affect STAT5; for example, the presence of HPV or the E7 oncoprotein promotes an increase in STAT5 phosphorylation, which is essential for the amplification of the viral genome [210]. On the other hand, the inhibition of STAT5b in HeLa (HPV18) and CasKi (HPV16) cervical cancer cells induces a reduction in proliferation, in the colony formation capacity, in the expression of cyclin D1, and in the anti-apoptotic protein Bcl-xL, which correlates with an increase in the expression of the cell cycle regulatory protein p21, the presence of cleaved PARP, and an increase in apoptosis. These data imply the relevance of STAT5b in cervical cancer. It is important to note that JAK2 or STAT3 inhibition show similar behaviors [190,208].

STAT6 comprises 850 amino acids, with tyrosine 641 as its phosphorylation site [211]; however, S407 could be the critical phosphorylation point for the total activation of STAT6 in response to a viral infection [212]. STAT6 participates mainly in transducing IL-4 and IL-13 signals [213]. STAT6 activation induced by IL-4 is essential to Th2 cell differentiation and immunoglobulin isotypes conversion [214,215,216]. STAT6 promotes different functions, such as the proliferation and maturation of B cells, mediates the expression of MHC-II and IgE, and plays an essential function in the activation of mast cells [217]. The role of STAT6 in cervical cancer is poorly studied, and very few reports indicate that HeLa cells express STAT6 constitutively and that a small proportion is in its phosphorylated form. Zhang’s group in 2017 showed that expression of HPV-16 E6 and E7 in non-small lung cancer cells increased levels of phosphorylated STAT6. On the other hand, Li et al., in 2013, showed that the treatment of HeLa cells with co-immobilized IFN-γ/TNF-α nanoparticles promoted an increase in the expression of STAT6 and its phosphorylation, the induction of apoptosis, the expression of p53 and Bax and the decrease of Bcl-2. However, when they treated silenced-STAT6 (shSTAT6) HeLa cells with co-immobilized IFN-γ/TNF-α nanoparticles, they observed the opposite effect, a decrease in apoptosis and the expression of p53 and Bax, in addition to an increase in the antiapoptotic protein Bcl-2 [218,219]. These data show the importance of studying STAT6 in the induction of cell death in cervical cancer.

## 5. Negative Regulators

Many negative regulators participate in regulating the JAK/STAT signal transduction. These regulators keep the balance and steady state of the JAK/STAT pathway. Several reports have shown that there are different mechanisms to negatively regulate the JAK/STAT signaling: suppressors of cytokine signaling (SOCS/CIS family), protein inhibitor of activated STAT (PIAS), and protein tyrosine phosphatases (PTPs) (Figure 1) [104].

### 5.1. SOCS/CIS Family

The SOCS protein family includes eight elements: CIS, SOCS1, SOCS2, SOCS3, SOCS4, SOCS5, SOCS6, and SOCS7. Activated STATs induce the expression of SOCS proteins, which bind to the complex of phosphorylated JAK and its cognate receptor to control the JAK/STAT pathway negatively. The SOCS family controls the JAK/STAT signaling pathway by different mechanisms: (a) SOCS proteins bind to the phosphotyrosine residue (pTyr) on the cell membrane receptor to interfere with the binding of the STAT protein [220]; direct and specific binding to the kinase or its receptor inhibits the catalytic activity of the JAK kinase [221,222]. For instance, overexpression of SOCS3 interferes with the activity of STAT5; also, SOCS3 can inhibit the Th1 response of the immune cells and stimulates a Th2 response by inhibiting the activity of STAT4 in response to IL-12 [223]. (b) SOCS proteins bind to JAK and STAT proteins and can induce their degradation in the proteasome. An elongation complex is formed by SOCS, the elongation protein BC, the cullin5-scaffold protein, and a ubiquitin-linked enzyme. This polyubiquitinated protein complex allows the proteasome to degrade JAKs and STATs binding to SOCS. This process can control the signals induced by the JAK/STAT pathway [224]. In precancerous lesions and different stages of cervical cancer, an undetectable or reduced expression of SOCS1 was reported compared to the expression in the normal cervix. In addition, Sobti’s group, in 2011, associated this decrease with the severity of the lesions [225]. Later, in 2015, Kim’s group analyzed SOCS1, SOCS3, and SOCS5 in cervical cancer samples and HPV+ cervical cancer cell lines; they found that the expression of these three SOCS proteins decreased compared to tissues from the normal cervix. They report that the decrease in the expression of SOCS1 is due to the inactivation of its gene by hypermethylation of its promoter and by histone acetylation. In contrast, the inhibition in the expression of SOCS3 is regulated only by histone acetylation. However, they also show that overexpression of SOCS1 or SOCS3 confers radioresistance to cervical cancer cells [226]. On the other hand, Kamio’s group in 2004 reported that the constitutive expression of SOCS1 in HeLa and CasKi cell lines inhibits proliferation, arrests the cell cycle, decreases the expression of the E7 oncoprotein, increases the amount of Rb protein, and inhibits the tumorigenicity, in addition to decreasing STAT3 phosphorylation. SOCS1 is essential in inhibiting the tumor transformation process because it can bind to the HPVE7 oncoprotein and induce its ubiquitination and proteasomal degradation [227].

### 5.2. PIAS

The PIAS family includes four proteins in mammals: PIAS1 (PIASx), PIAS2, PIAS3, and PIAS4 (PIASy). They have different splice variants: two splice variants for PIAS1 (PIASx-α and PIASx-β), one splice variant for PIAS3 (PIAS3b), and one splice variant for PIAS4 (PIASyE6) [228]. PIAS members are specific STAT inhibitors since specific PIAS can interact with specific STATs. For example, PIAS1 and PIAS4 usually bind to STAT1, while PIAS3 and PIAS1 typically bind to STAT3 and STAT4. PIAS proteins cannot interact with STAT monomers; thus, STATs must be tyrosine phosphorylated by JAKs, and then STAT dimers can interact with PIAS [104]. The PIAS family regulates signal transduction of the JAK/STAT pathway by inhibiting the DNA-binding activity of transcription factors [229,230], promoting transcription factor sumoylation [231], recruiting histone deacetylases to avoid STAT proteins binding to DNA, thus failing to induce the transcription of target genes [232], and chelating transcription factors to form repressor complexes to negatively regulate transcription [233].

### 5.3. PTPs

Protein tyrosine phosphatases (PTPs) function as tyrosine-specific or dual-specific and dephosphorylate both tyrosine and serine/threonine residues. The SH domain in protein PTPs functions as a docking site to interact with different signal transductors, activated cell membrane receptors, and JAK proteins to hydrolyze their phosphate group. Moreover, PTPs can remove phosphate groups from STATs to inhibit their activity, thus inhibiting the JAK/STAT signal transduction [234]. SH2-containing protein tyrosine phosphatase (SHP-1) is an essential element of the PTP family. Different receptors may activate SHP1; once it is activated, SHP-1 translocates to the nucleus, where it dephosphorylates STAT5 [235]. PTPs can also remove phosphates from JAK proteins; if JAKs are inactive, the JAK/STAT signaling pathway can be regulated. CD45 is a transmembrane PTP that inhibits the phosphorylation of JAK2 induced by IL-3 and negatively regulates JAK/STAT signal transduction, thereby inhibiting IL-3-mediated cell proliferation [236]. The non-receptor PTP1B mainly dephosphorylates STAT5 in order to regulate the JAK/STAT signal transduction cascades; nevertheless, it can remove phosphate groups from JAK2 and TYK2 from specific tyrosine motifs in the activation loop of the kinase domain of these proteins in the cytoplasm of the cell [237]. PTP1C binds via its SH2 domain to pY429 in the cytoplasmic region of the EPOR to remove phosphate groups from JAK2, negatively regulating its activity to terminate proliferative signals [238]. SH2-containing protein tyrosine phosphatase-2 (SHP-2) can negatively regulate STATs to avoid the cytotoxic effect of IFN to promote cell growth [239].

## 6. Signaling Pathways and HPV

HPVs have a double-stranded circular DNA enclosed in a capsid with an icosahedral structure that preferentially infect basal epithelial cells. A small group of HPVs are classified as high risk (hrHPVs) because they express the oncogenes E6 and E7 required to inhibit the immune system response and to deregulate other cellular processes such as proliferation. E6 and E7 oncoproteins of hrHPVs are usually found in precursor lesions and advanced stages of the tumor and can be considered tumor-specific antigens [240].

**Figure 3:**
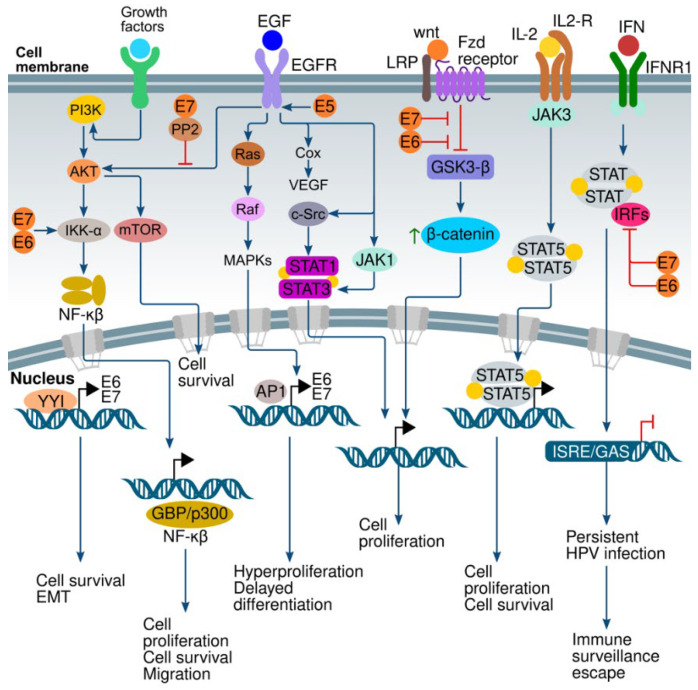
Different signaling pathways implicated in HPV-associated cancer. Cervical cancer is associated with HPV pathogenesis involved in the loss of normal control of key molecular processes that affect the proliferation, survival, migration, epithelial–mesenchymal transition (EMT), and immune surveillance escape of cancer cells.

Signal transduction pathways are the molecular mechanisms by which cells transduce an extracellular stimulus to the cytoplasm to control the transcription of genes that can regulate a wide variety of biological effects [241]. The tumor viruses (like HPV) manipulate many different signaling pathways to induce oncogenesis; this phenomenon initiates diverse cellular responses, which lead to the immortalization and proliferation of the infected cells [242]. HPV virus can activate several signal transduction cascades that are implicated in the release of the normal control of critical molecular processes that affect cell proliferation and differentiation. Here, we describe and discuss various signal transduction pathways deregulated by HPV (Figure 3).

### 6.1. Interferon (IFN) Pathway

The interferon pathway is a central component of the innate immune system that senses the presence of DNA in the cells’ cytosol and triggers the activation of defense mechanisms through IFN pathways. Interferons (IFNs) are very active cytokines which play a central role in eliminating pathogen infections by controlling inflammation and immune response. HPVs are DNA viruses; thus, the IFN pathway directly induces anti-virus molecular countermeasures by the cyclic GMP–AMP synthase (cGAS)–stimulator of interferon genes (STING) pathway (cGAS-STING). The activation of cGAS after DNA binding generates cyclic GMP–AMP (cGAMP), which is a second messenger that binds to the adaptor protein STING located in the endoplasmic reticulum [243,244]. HPV oncoproteins can support persistent infection, counteract the cGAS/STING/IRF3 axis, and activate interferon-stimulated gene (ISG) responses [245,246,247]. Viral proteins E2 and E6 can downregulate the expression of STING and IFNκ [248,249]. The inhibition of IFNκ expression downregulates ISGs transcription and could play a critical role by promoting tumors induced by HPVs [248].

E6 oncoprotein from HPV-16 binds to interferon regulatory factor (IRF) 3, thus suppressing its transcriptional activity [250]. E7 oncoprotein from HPV-16 represses the transcription of IFNβ by binding to IRF1 and recruiting histone deacetylases (HDACs) to the promoter [251,252]. HrHPV synergized with hyperactivated yes-associated protein 1 (YAP1) (from HIPPO/YAP1 pathway) to promote the initiation and progression of cervical cancer. The hyperactivation of YAP1 oncogene in cervical epithelial cells restrained the activation of transcription factors such as IRF1, IRF3, and IRF7, decreasing IFNA1, IFNB1, and IFNE, which are necessary for the production of type I IFNs in primary cultures of human cervical epithelial cells. He et al. have shown that when the Hippo pathway is interrupted, subsequent activation of YAP1 oncogene in the cervical epithelia leads to an alteration in the innate antiviral immunity, allowing HPV to escape immune surveillance, ending in persistent HPV infection [253].

It is important to note that activation of the IFN pathway is essential in the response to HPV in cervical cancer. Some studies show that the treatment of HPV18 tumor cells HeLa with IFN-a (Type I) induces a slight decrease in their proliferation and apoptosis induction, while the treatment of SiHa (HPV16) and HeLa with IFN-γ (Type II) induces autophagy [254,255]. Concerning the effect of IFN-type III on cervical cancer, there is only one report showing that low-risk HPV-positive cervical cells express higher levels of the IFN lambda1 and IFN-lambdaR1 genes compared with hrHPV-positive cervical cells and HPV-negative cervical cells, suggesting a likely mechanism for the IFN-type III response orchestrated by hrHPVs [256].

### 6.2. Wnt/β-Catenin Pathway

Activation of the Wnt/β-catenin pathway is essential to establishing the transformation and immortalization of HPV-positive epithelial cells and maintain tissue homeostasis and pathologic phenotype [257]. E6 oncoproteins of high- and low-risk HPVs can activate the Wnt/β-catenin pathway [258,259]. This signaling pathway plays an essential role in cell proliferation and differentiation, as well as deregulation of the WNT/β-catenin pathway leads to many types of human cancers [260,261]. Abnormal activation of the Wnt pathway by disabled negative regulators or by overexpression of activators (Wnt-ligands) leads to the inhibition of GSK3-β which causes accumulation of β-catenin in the cytoplasm. β-catenin translocates to the nucleus, where it forms an active transcriptional complex and induces the transcription of genes such as c-Jun, cyclin D, Axin2, survivin, vascular endothelial growth factor (VEGF), COX-2, c-myc, and matrix metalloproteinase-7 (MMP-7) [260,262,263].

### 6.3. PI3K/AKT/mTOR Pathway

The PI3K/Akt/mTOR pathway is essential for cellular control and signal transduction: it stimulates cell survival, growth, and proliferation, prevents apoptosis, and induces migration and energy metabolism reprogramming [264,265]. The activated PI3K/Akt pathway responsible for signal transduction from the cell surface to the nucleus is a main cancer survival pathway [261]. Many reports demonstrate that PI3K signals are activated and amplified in HPV-positive cervical cancers [266]. Moreover, mutations in PIK3CA (the gene that codifies class I catalytic subunit) and PTEN (phosphatase and tensin homolog, is a negative regulator of PI3K pathway) are more common in HPV-positive than HPV-negative head and neck squamous cell carcinomas (HNSCC) [267,268]. The HPV-16 E7 oncoprotein attaches to protein phosphatase 2A (PP2A) subunits, interfering with p-Akt interaction, thus avoiding its inactivation. This interaction explains how E7 oncoprotein increases AKT activity and correlates with its capacity to disable Rb protein, leading to intraepithelial lesions (high grade) [269]. E6 and E7 oncoproteins can activate Akt or bind tuberous sclerosis 2 (TSC2), leading to its degradation, and as a result, it stimulates mTORC1 [270,271,271,272].

### 6.4. ERK/MAPK Pathway

The extracellular signal-regulated kinase (ERK) signal transduction pathway is positioned downstream of different growth factors, cytokines, and hormones; it activates various substrates involved in different cell responses such as proliferation, differentiation, survival, and motility, and plays a critical role in regulating tumorigenesis [273]. The p21Ras (Rat sarcoma) GTPase recruits RAF1 (Rapidly Accelerated Fibrosarcoma), which phosphorylates specific serine residues of MEK1/2 (MAPK/ERK kinase 1 and 2) [274,275]. MEK1/2 phosphorylates specific tyrosine and threonine residues of ERK1/2 kinase to activate several downstream signaling cascades [273,276]. On the other hand, ERK1 expression is an early marker for cervical cancer [277]. Some reports have shown that HPVE5 can promote cell proliferation and activate the MAPK/ERK cascade to activate their target transcription factors, including Ets1/2, Elk-1, c-fos, c-myc, and c-jun [278,279,280,281]. E5 protein induces the expression of VEGF by activating ERK; this affects the regulation of the phosphorylation of ERK so that E5 stabilizes VEGF. The HPV-infected cells are resistant to autophagy and apoptosis activation due to the presence of HPVE5 protein and the constant activation of the MAPK-ERK signaling cascades [282,283,284]. Liu et al. reported that HPV-16 E6 oncoprotein induced HIF-1α, VEGF, and IL-8 expression, having, as a result, enhanced angiogenesis in non-small cell lung cancer (NSCLC) cells via ERK1/2 [285]. 

### 6.5. Ying and Yang 1 (YY1) Pathway

Dysregulated epigenetic pathways can be associated with cancer development. Transcription factor YY1 belongs to the polycomb group protein family. This protein has a zinc finger motif to bind to DNA; it functions as a repressor, activator, or an initiator element-binding protein of the transcriptional control of different genomes depending upon the context in which it binds; therefore, it is considered a master regulator of the transcription of several cellular and viral genes. Transcription factor YY1 plays an essential role in the epigenetic regulation of transcription and participates in stem-cell identity, differentiation, survival, metastasis, and resistance to chemotherapy [286,287]. A few growth factors can stimulate the expression of the YY1 gene, whilst antiproliferative signals inhibit its expression [288,289]. YY1 affects the LCRs of HPV-16 and -18 and regulates HPV E6 and E7 transcription [290]; furthermore, YY1 can bind to regulatory regions of HPV to regulate the transcription of viral oncogenes and can regulate multiple cellular functions because it contains different activator and repressor domains recognized by YY1 [291,292]. For example, UCRBP, NF-E1, and CF1 are some targets of YY-1 and can regulate E6 and E7 oncogenes and helps to maintain HPV infection [293,294]. The suppression of YY1 induces activation of p53 and apoptosis in the cervical cancer cell line HeLa [294]. In cervical carcinomas, YY1 is overexpressed and is essential in the progression of HPV-infected cervical carcinomas. Moreover, inhibition of p53 and As_2_O_3_-induced apoptosis activated by YY1 were detected in HPV-infected cervical cancer cells; thus, this protein could be an effective target for HPV-positive cervical cancer treatment [294].

### 6.6. EGFR Family Pathway

The epidermal growth factor receptor (EGFR/HER1) belongs to the ErbB/HER receptor family; the other members are ErbB2/HER2/neu, ErbB3/HER3, and ErbB4/HER4 [295]. This family of receptors are transmembrane proteins with tyrosine kinase activity, which are activated by the binding of different extracellular ligands; for example, epithelial growth factor (EGF), the transforming growth factor α (TGF α), and neuregulin-1, among others [296]. The functional structure of these receptors includes an extracellular domain containing the ligand binding site, a transmembrane domain, and an intracellular domain containing the catalytic domain with kinase activity. The member model of the family is EGFR, a monomer; the binding of EGF activates the receptor, inducing the formation of homodimers. EGFR is a well-characterized receptor that activates signaling cascades that produce diverse cellular responses, including proliferation, survival, differentiation, migration, and angiogenesis [297,298,299]. After ligand binding, EGFR activates and forms homodimers that autophosphorylate specific tyrosine residues and promote extracellular mitogenic signals to the nucleus. Many cellular pathways are activated, such as the MAPK, the p21Ras, and the PI3K/Akt, which are implicated in proliferation, motility, and survival [297]. EGFR has a central role in cancer cell proliferation: it can directly regulate diverse metabolic processes, including glucose catabolism and fatty acids and pyrimidines synthesis, by activating enzymes regulated by phosphorylation or by crosstalking to different signaling pathways, such as the Akt pathway [300,301,302]. 

High expression of EGFR is associated with poor outcomes in cervical cancer [303]. The HPV oncoprotein E5 activates and increases the EGFR pathway depending on the ligand [304]. HPV E5 can upregulate VEGF (vascular endothelial growth factor) and cyclooxygenase 2 through EGFR [305]. Moreover, E5 can activate the EGFR pathway to induce a cytoplasmic molecular pathway that activates different proto-oncogenes. For example, MAP kinases and the activating protein-1 (AP-1) are constitutively activated, sending increased signals to activate the transcription of the viral oncoproteins E6/E7 [304,306]. Furthermore, HPV-16 E5 increases the recycling of the EGFR to the cell surface and, consequently, the phosphorylation of EGFR augments, although the binding of its ligand is still needed [307,308]. E5 induces an increased proliferation of keratinocytes, amplifying EGFR signaling to delay cell differentiation [304]. It has been shown that an increased EGFR expression correlates with low survival rates and radioresistance. When EGFR is inhibited, cancer cells show a higher sensitivity to ionizing radiation in preclinical studies on HNSCC [309,310,311]. It has been reported that the EGFR present in cervical cancer cell lines CALO and INBL has putative mutations in the region αC of the kinase domain, resulting in EGFR being present but not phosphorylated [312]. 

Other family members are deregulated in HPV-infected cells; for example, it has been shown that HPV-positive tumors have a high expression of HER2 and HER3 receptors [313,314]. It has been reported that HER2 expression in recurrent advanced cervical tumors is often related to poor outcomes [315]. Some studies, including whole exome sequencing analysis, found that HER2 is constitutively active due to somatic mutation, amplification and the presence of HPV integration sites close to the ERBB2 gene in cervical cancer [316]. There is an association between HER3 overexpression and HPV infection in head and neck cancers which could be related to a poor survival rate. Other studies found that HER3 expression is regulated by HPV E6/E7 oncoproteins and is associated with downstream PI3K signaling, which strongly suggests an association between HER3 expression and HPV-associated cancer [317].

### 6.7. NF-κB Pathway

The family of transcription factors, nuclear factor-kappa B (NF-κB), consist of five members, but the foremost is a heterodimer formed by p65 (RelA) and p50 subunits. These dimers are inactive when they interact with the inhibitor of nuclear factor kappa B (IκB); they are released from IκB in response to different stimuli that activate NF-κB to translocate to the nucleus [318]. NF-κB is a pleiotropic transcription factor with essential roles in innate immunity, inflammation, differentiation, viral replication, and tumorigenesis [319,320]. The canonical pathway is initiated by external stimuli such as antigens, growth factors, or cytokines bound to their respective receptors. However, it depends on IκB release by the enzyme complex that includes IκB kinases (IKK) and the accessory protein NEMO (NF-κB essential modulator) [321]. NF-κB is implicated in the development of several cancers, and it plays an essential role in controlling different gene functions to induce responses such as cell proliferation, migration, angiogenesis, and apoptosis. It has been shown that cervical cancer progression is associated with a high expression of NF-κB and its enhanced DNA binding activity [322]. Mishra et al. showed the common presence of the homodimer p50/p50, mostly in HPV tumors. In contrast, the presence of p65 was more abundant in HPV infection, increasing differentiation in head and neck cancer cells with better outcomes for the patients [323]. Selective crosstalk between the heterodimer NF-kB/c-Rel with AP-1/Fra-2 induced an aggressive tumor phenotype and poor prognosis, mainly in patients with HPV-negative tongue squamous cell carcinoma (TSCC). On the contrary, patients with HPV-positive TSCC had a better prognosis because the viral infection increased the expression of p65, p27, and Fra-2, which induced cell differentiation [324].

In the case of cervical cancer, the presence of E6/E7 oncoproteins affects the activity of NF-κB, leading to an inadequate immune response [325]. As a consequence, the viral infection cannot be cleared, and, if it persists, can develop into cancer. NF-κB is reactivated after the cancerous lesions are formed in response to the cytokines released by M2 macrophages in the tumor microenvironment [321,326,327]. Moreover, the persistent infection can induce mutations in molecules, such as EGFR or RAS, which are upstream in the cascade of NF-κB, affecting its function. This altered activity induces the expression of genes, such as telomerase and c-myc, among others, which induce cell immortalization and proliferation, as well as metastasis (epithelial-mesenchymal transition) and angiogenesis [318,328]. Its reactivation induces expression of the AID/APOBEC (activation-induced cytokine deaminase) protein family known to participate in cancer development by causing genomic damage [329]. 

### 6.8. miRNAs

Micro ribonucleic acids (miRNAs) are small non-protein-coding single-strand RNAs, 18–25 nucleotides in length, and participate in mRNA translation, therefore regulating gene expression. miRNAs have a central role in regulating multiple cellular responses, such as cell growth, proliferation, differentiation, apoptosis, cell migration, and metastasis. Since miRNAs are non-coding, they modulate target gene expression by binding to RNA to degrade its gene target or inhibit target gene translation. They interfere with RNA and can have opposite functions by altering its expression towards an oncogenic or tumor suppressor function [330]. Some of the main miRNAs expressed in HPV-infected cells are miR-21 and miR-7a, which are upregulated in solid tumors, and have a central role in oncogenic signaling. They are transcriptionally induced by AP-1, which is essential for HPV transcription maintaining STAT3 activated in HPV-infected cells [331]. miR-29, often downregulated, prevents cell cycle progression, induces apoptosis, and promotes malignant transformation induced by HPV [332,333]. miR-218 can upregulate the expression of epithelial cell-specific marker LAMB3 through the PI3K/Akt pathway [334,335]. miR-34a expression is downregulated via E6/p53 and is associated with hrHPV infection and cervical cancer develop [336,337]. Expression of Hsa-miR-139-3p is frequently downregulated in HPV-infected cells, and Sannigrahi et al. found that upon upregulation of this miRNA, it restores p53 function, and chemoresistance can be reverted and inhibits E6/E7 [338]. miRNAs are key regulators in cancer development. It is vital to find more specific miRNAs for cervical cancer that can help in specific diagnoses and to develop a miRNA-based therapy.

## 7. Conclusions

In the past thirty years, significant advances have helped us to understand the initial transformation steps and the carcinogenesis process associated with HPV oncoproteins in the cervix. However, recurring or persistent cervical cancer represents a public health problem and is a significant cause of death related to this cancer in non-developed countries. Thus, it is necessary to fully understand the molecular mechanisms associated with cervical tumorigenesis to establish rapid diagnostic methods and more effective treatments for cervical cancer. The JAK/STAT signaling pathway is involved in cell surface receptor-mediated signal transduction that accounts for diverse responses to extracellular signaling molecules and is implicated in initiation, progression, metastasis, and resistance to treatment of cervical cancer. Soon, technological advances will help us understand this critical pathway for cervical cancer development, including cell-extrinsic and cell-intrinsic factors that regulate JAK/STAT activity, as well as the molecular mechanisms of dysregulated JAK/STAT signaling which could help us find specific targets to inhibit the pathway, leading to more efficient and less severe cancer treatments.

## Figures and Tables

**Table 1:** DNA binding motif sequence of STATs and their DNA target.

	DBD Sequence (Protein)	Optimal Binding Sequence (DNA) [131]
STAT1	317-FVVERQPCMPTHPQRPLVLKTGVQFTVKLRLLVKLQELNYNLKVKVLFDKDVNERNTV KGFRKFNILGTHTKVMNMEESTNGSLAAEFRHLQLKEQKNAGTRTNEGPLIVTEELHSL SFETQLCQPGLVIDLETTSLPVVVISNVSQLPSGWASILWYNML-437	TTCCCGTA
STAT2	312-FVVETQPCMPQTPHRPLILKTGSKFTVRTRLLVRLQEGNESLTVEVSIDRNPPQL QGFRKFNILTSNQKTLTPEKGQSQGLIWDFGYLTLVEQRSGGSGKGSNKGPLGV TEELHIISFTVKYTYQGLKQELKTDTLPVVIISNMNQLSIAWASVLWFNLL-432	GGGAAACCGAAACTG
STAT3	321-FVVERQPCMPMHPDRPLVIKTGVQFTTKVRLLVKFPELNYQLKIKVCIDKDSGD VAALRGSRKFNILGTNTKVMNMEESNNGSLSAEFKHLTLREQRCGNGGRANC DASLIVTEELHLITFETEVYHQGLKIDLETHSLPVVVISNICQMPNAWASILWYN MLT-421	TTCCCGTAA
STAT4	316-FVVERQPCMPTHPQRPLVLKTLIQFTVKLRLLIKLPELNYQVKVKASIDKNVSTL SNRRFVLCGTNVKAMSIEESSNGSLSVEFRHLQPKEMKSSAGGKGNEGCHMVT EELHSITFETQICLYGLTIDLETSSLPVVMISNVSQLPNAWASIIWYNVS-436	TTCCCAGAA
STAT5	332-FIIEKQPPQVLKTQTKFAATVRLLVGGKLNVHMNPPQVKATIISEQQAKSLLKN ENTRNECSGEILNNCCVMEYHQATGTLSAHFRNMSLKRIKRADRRGAESVTEE KFTVLFESQFSVGSNELVFQVKTLSLPVVVIVHGSQDHNATATVLWDNAFA-452	TTCCTGGAA
STAT6	273-FLVEKQPPQVLKTQTKFQAGVRFLLGLRFLGAPAKPPLVRADMVTEKQARELS VPQGPGAESTGEIINNTVPLENSIPGNCCSALFKNLLLKKIKRCERKGTESVTEEK CAVLFSASFTLGPGKLPIQLQALSLPLVVIVHGNQDNNAKATILWDNAF-393	TTCTGGAA

All STATs can bind to canonical STAT-binding site TTNNNNNAA.

## Data Availability

Not applicable.
